# Gestalt Perceptual Organization of Visual Stimuli Captures Attention Automatically: Electrophysiological Evidence

**DOI:** 10.3389/fnhum.2016.00446

**Published:** 2016-08-31

**Authors:** Francesco Marini, Carlo A. Marzi

**Affiliations:** ^1^Department of Neuroscience, Biomedicine and Movement Sciences, University of VeronaVerona, Italy; ^2^Department of Psychology, University of California San Diego, La JollaCA, USA; ^3^National Institute of NeuroscienceVerona, Italy

**Keywords:** gestalt, visual attention, automatic capture, ERP, N2pc

## Abstract

The visual system leverages organizational regularities of perceptual elements to create meaningful representations of the world. One clear example of such function, which has been formalized in the Gestalt psychology principles, is the perceptual grouping of simple visual elements (e.g., lines and arcs) into unitary objects (e.g., forms and shapes). The present study sought to characterize automatic attentional capture and related cognitive processing of Gestalt-like visual stimuli at the psychophysiological level by using event-related potentials (ERPs). We measured ERPs during a simple visual reaction time task with bilateral presentations of physically matched elements with or without a Gestalt organization. Results showed that Gestalt (vs. non-Gestalt) stimuli are characterized by a larger N2pc together with enhanced ERP amplitudes of non-lateralized components (N1, N2, P3) starting around 150 ms post-stimulus onset. Thus, we conclude that Gestalt stimuli capture attention automatically and entail characteristic psychophysiological signatures at both early and late processing stages.

Highlights

We studied the neural signatures of the automatic processes of visual attention elicited by Gestalt stimuli. We found that a reliable early correlate of attentional capture turned out to be the N2pc component. Perceptual and cognitive processing of Gestalt stimuli is associated with larger N1, N2, and P3

## Introduction

The human brain makes sense of the multitude of sensory stimuli in the environment by grouping them into meaningful representations such as forms or objects. Behavioral research has demonstrated that representations of global objects, which are constituted by grouped individual elements, may capture attention even when top-down attention is directed toward the local individual elements rather than toward the global configuration (Rauschenberger and Yantis, 2001). Moreover, visual object representations are not limited to situations in which such objects are attended or task-relevant and may take place also when they are unattended and task-irrelevant (Russell and Driver, 2005; Lamy et al., 2006; Müller et al., 2010). Therefore, top-down attention may not be required in order to form object representations, and grouping of perceptual elements to form meaningful percepts may occur relatively automatically and independently of top-down attentional selection. However, neurophysiological evidence of bottom-up, automatic capture of attention by visual elements grouped into meaningful representations is still lacking. In this study, the event-related potentials (ERPs) technique was used to investigate whether the grouping of independent perceptual elements may elicit stimulus-driven attentional capture. Crucially, because we were interested in truly automatic processes, we used a simple detection task in which participants were required to detect the onset of any visual stimulus and respond as quickly as possibly by pressing a key without having to discriminate the stimuli or to make a choice. We adopted this simple detection task in order to avoid any top-down attentional bias, such as those due to target-template matching. This aspect represents a substantial difference relative to typical visual search tasks (pop-out or conjunction) and ensures that any attentional capture reflects genuinely automatic processes.

Several visual ERP components are modulated by attentional selection, both voluntary and automatic (or stimulus-driven). These ERP modulations start as early as 80 ms post-stimulus onset in the occipital cortex in the form of lateralized biases on the P1 and N1 components (Mangun, 1995; Hillyard and Anllo-Vento, 1998; Luck et al., 2000). ERP signatures of attentional capture by task-relevant and/or salient stimuli emerge in the parieto-occipital (PO) cortex around 200–250 ms (N2pc component; e.g., Luck and Hillyard, 1994). Afterward, late non-lateralized attentional ERP enhancements extend to the parietal and frontal cortices after 250 ms post-stimulus onset (P3 component; Kutas et al., 1977; Kok, 2001; Polich, 2007).

The overarching aim of the study was to characterize the psychophysiological and cognitive processing of visual stimuli organized according to specific grouping principles – operationalized here as the Gestalt principles of *Closure* and G*ood Form* (Banerjee, 1994; Wolfe et al., 2008; Wagemans et al., 2012). Previous ERP studies of visual stimuli with Gestalt organization showed that modulations in the visual cortex arise about 100 ms post-stimulus, but strongly decrease outside the area of selective attention (Han et al., 2002; 2005). Other ERP studies used the Kanizsa figure with bilateral displays and tasks in which participants were required to identify the illusory figure (target) while ignoring a contralateral distractor. An enhanced N2pc contralateral to the target hemifield was observed, indicating the attentional selection of the target stimulus and the concurrent suppression of the distractor (Conci et al., 2006; 2011; Wiegand et al., 2015). Whether or not similar results would be obtained when subjects are not required to perform a choice task, thereby reflecting automatic attentional capture rather than target selection, remains an open question.

A study that used bilateral square stimuli that could be either connected by a line or not found that connected squares were associated with a spread of attention across hemispheres, as indicated by reduced contralateral vs. ipsilateral differences in the N2pc amplitude for connected objects (Kasai and Kondo, 2007). Similar findings were obtained when bilateral stimuli were grouped based on feature similarity rather than on their connectedness (Kasai et al., 2011). These studies showed reductions of attention-related lateralized ERP patterns when bilateral objects were grouped together based on connectedness or feature similarity. Differently, here we were interested in investigating the attentional competition between bilateral stimuli when one of them is perceptually organized according to Gestalt grouping principles and the other is not, by using a simple detection task. We hypothesized that simple visual elements such as lines and arcs, when organized according to Gestalt grouping principles, exhibit an inherent saliency and tend to capture attention automatically. Accordingly, we reasoned that visual processing and attentional orienting processes might differ between Gestalt-like stimuli (hereinafter, *Gestalt stimuli*) and non-Gestalt-like stimuli (hereinafter, *non*-*Gestalt stimuli*) to reflect this putative saliency of Gestalt stimuli, and made two specific hypotheses: (i) Gestalt stimuli are associated with a larger attentional capture observed contralaterally about 250 ms post-stimulus onset with respect to non-Gestalt stimuli, and (ii) the perceptual and attentional (non-lateralized) processing of Gestalt stimuli entails enhanced electrophysiological signatures from early throughout late processing stages.

In order to test these hypotheses, we devised an experimental design in which two visual stimuli were presented bilaterally on each trial (one stimulus per hemifield), but differently from previous work (Kasai et al., 2011) sensory information was fully balanced across hemispheres. As a test of the first hypothesis, on half of the trials a Gestalt stimulus in one hemifield (left or right) was presented with a non-Gestalt stimulus in the opposite hemifield (right or left, respectively). The bilateral presentation of Gestalt and non-Gestalt stimuli on each of these trials should elicit a competition for the deployment of lateralized attentional resources between the two types of configurations. On these trials, we were interested in an electrophysiological signature of the attentional capture elicited by lateralized salient stimuli, the N2pc, i.e., a negative component of the ERP waveform that is typically observed contralateral to the side of attention-capturing stimuli (Luck and Hillyard, 1994; Eimer, 1996; Girelli and Luck, 1997; Hickey et al., 2006). Therefore, in line with our hypothesis of stimulus-driven capture exerted by Gestalt stimuli, we expected to observe a larger N2pc contralateral to Gestalt (vs. non-Gestalt) stimuli.

The second hypothesis was tested on the remaining half of the trials by presenting in each hemifield identical visual configurations of the same type (either two Gestalt or two non-Gestalt stimuli) thus presumably eliciting a non-lateralized ERP response characteristic of the type of configuration. On these trials, we were interested in non-lateralized visual ERPs, at early (P1, N1), mid-level (N2), and late (P3) latencies. As to the contrast bilateral Gestalt versus bilateral non-Gestalt trials, we predicted that Gestalt stimuli: (i) would show an advantage in early attentional processing relative to non-Gestalt stimuli, as might be indicated by a larger amplitude of the N1 component; (ii) would be associated with an increased cognitive processing and attentional engagement, possibly leading to larger amplitude of the P3 component.

## Materials and Methods

### Participants

The sample size of 15 participants was determined *a priori* assuming 80% power and a large effect size (Cohen’s *d_z_* = 0.8) based on the critical N2pc analysis (paired *t*-test). Two participants of the original sample were excluded and then replaced because a high number of trials (>20%) showed EEG artifacts due to eye movements and/or blinks. The analysis was conducted on data from 15 participants (mean age ±*SD*: 27.3 ± 7.7, 8 females, all right-handed). All participants gave written informed consent to participate in the study and were compensated for their time at the rate of 12 Euro/h. The research was conducted in agreement with the principles of the Declaration of Helsinki (World Medical Association, 1996) and was approved by the Ethics Committee of the Verona “Azienda Ospedaliera Universitaria Integrata” (Codice Protocollo CAM-13) and by the ERCEA Committee (Proposal 339939 “Perceptual Awareness”).

### Experimental Design

**FIGURE 1:**
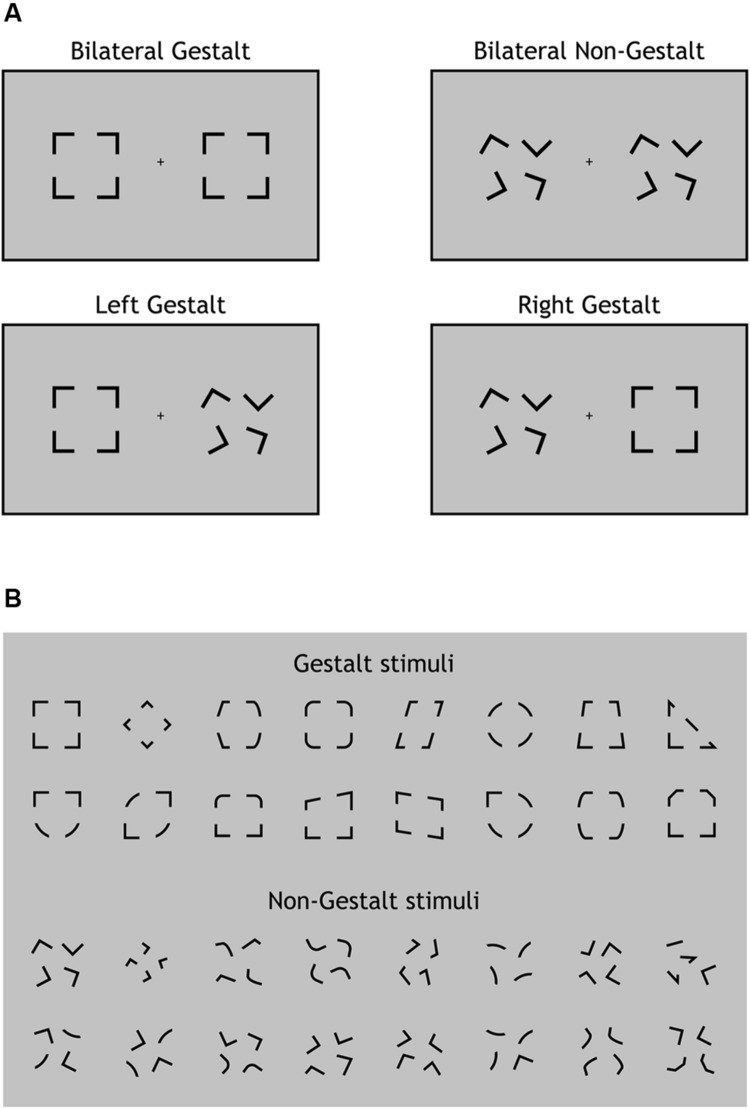
**Experimental design and stimuli. (A)** The four types of visual displays used in this study. On each trial, visual stimuli were presented bilaterally (one per hemifield). A Gestalt stimulus configuration could be present for both stimuli (Bilateral Gestalt condition, *GG*), for none of the stimuli (Bilateral Non-Gestalt, *nn*), only for the left stimulus (Left Gestalt, *Gn*), or only for the right stimulus (Right Gestalt, *nG*). **(B)** The 16 different configurations used for Gestalt (top) and Non-Gestalt (bottom) stimuli throughout the study.

The experimental paradigm was programmed in Matlab (MathWorks, Inc.) using Psychtoolbox 3 (Kleiner et al., 2007). Visual stimuli were presented in black (luminance 0 cd/m^2^) on a 19-inch monitor (Philips 109B, resolution: 1024 × 768, refresh rate: 60 Hz) with a uniform gray background (luminance 29.9 cd/m^2^) and auditory stimuli were presented through two loudspeakers located on the left and right side of the monitor, respectively. Visual stimuli were constituted by two visual configurations presented simultaneously for 83 ms one in the left and the other in the right hemifield along the horizontal meridian. The width of each configuration was 4.5° of visual angle and the lateral distance from the central fixation point to the innermost side of each configuration was 4°. Visual configurations were extracted from two ad-hoc groups of 16 configurations each characterized by being spatially organized according to the Gestalt principles of *Closure* and *Good Form* (e.g., Wolfe et al., 2008) (**Figure 1B**, upper part) or not (**Figure 1B**, lower part). Gestalt and non-Gestalt stimuli contained the same perceptual information, yet they differed for their configuration since all non-Gestalt configurations were non-symmetrical while most Gestalt configurations were symmetrical (**Figure 1B**).

Stimuli on each trial were randomly chosen (with equal frequency) among four possible types of displays: (i) Gestalt-like configuration on both sides (Bilateral Gestalt condition, *GG*; **Figure 1A**, upper left); (ii) non-Gestalt-like configuration on both sides (No Gestalt condition, *nn*; **Figure 1A**, upper right); (iii) Gestalt-like configuration in the left hemifield and non-Gestalt-like configuration in the right hemifield (Left Gestalt condition, *Gn*; **Figure 1A**, lower left); (iv) non-Gestalt-like configuration in the left hemifield and Gestalt-like configuration in the right hemifield (Right Gestalt condition, *nG*; **Figure 1A**, lower right). We will refer to *GG* and *nn* conditions as *bilateral Gestalt* and *bilateral non-Gestalt* conditions, respectively, while we will refer to *Gn* and *nG* conditions as *unilateral Gestalt* conditions. Bilateral Gestalt conditions (*GG* and *nn*) were contrasted in the ERP analysis of non-lateralized components (P1, N1, N2, P3), while unilateral Gestalt conditions (*Gn* and *nG*) were used for investigating within-trial lateralized ERP modulations as indexed by the N2pc component.

On each trial, prior to the presentation of the visual stimulus (inter-trial interval: 800–1200 ms, jittered), a sine-wave tone (duration: 250 ms, frequency: 587 Hz) alerted participants of an upcoming trial. On about 15% of trials, the tone was not followed by any visual stimulus (“catch trials”). On each trial, except catch trials, participants were required to press a key with the index finger of their right or left hand (randomized across subjects) as quickly as possible whenever they detected any visual stimulus. Participants were given 1500 ms after stimulus onset to carry out their response. It is worth emphasizing that the task did not require any discrimination; rather, this was a simple detection task, and therefore the Gestalt versus non-Gestalt characteristics of the stimuli were completely irrelevant as to response requirements. The experiment included 128 trials per condition and an additional 96 catch trials (608 total trials per subject). The duration of the experiment was about 45 min.

### EEG Recordings

EEG was recorded by using a 32-channel Brain Vision amplifier and an ElectroCap cap with Ag/AgCl electrodes placed at 28 scalp sites (Fp1, Fp2, F3, F4, T7, C3, Cz, C4, T8, CP5, CP1, CP2, CP6, P7, Pz, P8, PO7, PO3, PO4, PO8, O1, Oz, O2, I1, Iz, I2, according to the modified 10–20 system; Gilmore, 1994) and at the left and right mastoid. Electrooculogram (EOG) was recorded using three electrodes placed at the outer canthi of each eye and below the right eye, respectively. The EEG and EOG were low-pass filtered during acquisition (half-amplitude cutoff: 250 Hz) and digitized at 1 kHz.

Analyses were conducted with software packages EEGLAB (Delorme and Makeig, 2004) and ERPLAB (Lopez-Calderon and Luck, 2014). Signals were re-referenced oﬄine to the average of the mastoid electrodes and hi-pass filtered (half-amplitude cutoff: 0.1 Hz) for reducing low-frequency drifts. Epochs were created with a duration of 800 ms (starting 200 ms before stimulus onset and ending 600 ms after stimulus onset) and baseline correction was applied on the pre-stimulus interval (from -200 to 0 ms) and used for all analyses. Artifact detection was conducted by identifying epochs where either the EOG signal (re-referenced bipolarly) exceeded ±75 μV within a 200 ms moving window or any EEG channel exceeded ±200 μV within a 500 ms moving window.

All epochs with detected artifact were visually inspected prior to artifact rejection, which led to the exclusion of a proportion of epochs ranging between 2.2 and 8.8% across participants. Averaged ERP waveforms were computed for each subject and experimental condition, and then group averages were computed for each experimental condition (*GG*, *Gn*, *nG*, *nn*). For display purposes only, EEG waveforms were additionally filtered with a fourth-order low-pass filter (half-amplitude cutoff: 25 Hz, roll-off: 24 db/octave). EEG data for analysis purposes were not low-pass filtered.

### Analysis

Behavioral measures of response time (RT) and detection rates were extracted from the non-rejected trials using ERPLAB. Responses faster than 150 ms (anticipations) and slower than 600 ms (late responses) were considered outliers (on average, 0.5% of total trials) and excluded from the analysis. Importantly, trials excluded from the behavioral analysis were also excluded from the ERP analysis and vice-versa.

In order to obtain a measure of the response evoked by visual stimuli at the net of any influence of the preceding tone, ERPs relative to catch trials were subtracted from the group-averaged waveforms of each condition. The main dependent variable of the ERP analysis was the mean amplitude within specific time ranges and at electrode sites defined on the basis of the existing literature. The within-trial analysis of Gestalt configurations was conducted in the N2pc time range (220–300 ms post-stimulus onset, electrodes: PO7/8). The across-trial analysis of Gestalt configurations was conducted within four time windows: (i) the P1-range (90–150 ms, electrodes O1/2, PO7/8, P7/8); (II) the N1-range (150–210 ms, electrodes O1/2, PO7/8, P7/8); (iii) the N2-range (220–300 ms, electrodes O1/2, PO7/8, P7/8); (iv) the P3 range (250–350 ms, electrodes CP1/2, C3/4). The *a priori* power analysis was conducted using G-Power. Behavioral and EEG data were analyzed with paired *t*-tests and with fixed-effect repeated-measure analysis of variance (ANOVA) as implemented in the software Statistica 6.0 (StatSoft). In ANOVA, degrees of freedom were corrected for non-sphericity when appropriate.

## Results

### Behavior

**FIGURE 2:**
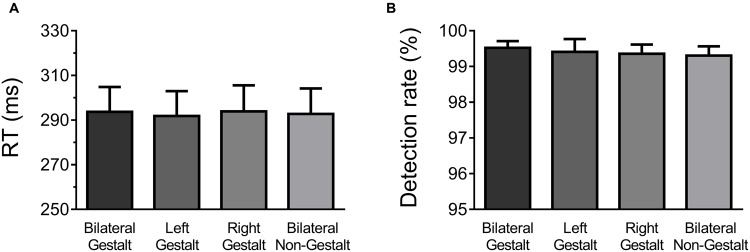
**Behavioral results. (A)** Mean response times (RT) and across-subject standard error of the mean for each experimental condition. **(B)** Mean detection rates and across-subject standard error of the mean for each experimental condition.

Behavioral data were analyzed with one-way ANOVAs factoring Experimental Condition (GG, Gn, nG, nn; **Figure 2A**). All conditions elicited very similar RTs (mean RTs ±*SD* per condition: GG = 299 ± 5 ms, Gn = 298 ± 5 ms, nG = 298 ± 5 ms, and nn = 298 ± 6 ms) and no significant differences were found [*F*(3,14) = 0.21, *p* = 0.89]. Likewise, no differences were observed in detection rates, which were at ceiling levels across all experimental conditions (mean Detection Rate in percentage ±*SD* per condition: GG = 99.5 ± 0.01, Gn = 99.4 ± 0.01, nG = 99.4 ± 0.01, and nn = 99.3 ± 0.01) [*F*(3,14) = 0.29, *p* = 0.83] (**Figure 2B**).

### EEG

#### Within-Trial Analysis of Gestalt Configurations (Gn and nG Trials)

##### N2pc

**FIGURE 3:**
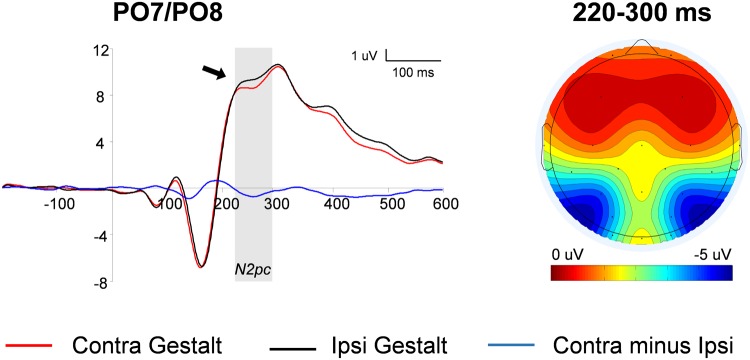
**N2pc analysis: waveforms and voltage maps.** Average ERP (event-related potentials) waveforms of the N2pc component measured at parieto-occipital (PO) locations contralateral to Gestalt stimuli (red line) and ipsilateral to Gestalt stimuli (i.e., contralateral to non-Gestalt; black line), on trials with unilateral Gestalt configuration (Gn and nG). The blue line represents the N2pc difference wave (red line minus black line). The arrow indicates a significant effect and the shaded gray area indicates the time-windows in which the significant effect was found. The map on the right shows the average voltage map relative to the N2pc difference wave within the 220–300 ms time-window.

The N2pc was computed on trials with unilateral Gestalt configurations (i.e., *Gn* and *nG* trials). First, waveforms contralateral to Gestalt configurations and waveforms contralateral to non-Gestalt configurations were collapsed across hemispheres. Then, the difference wave between the ERP contralateral to the Gestalt configuration minus the ERP ipsilateral to the Gestalt configuration was calculated. The contrast between these two conditions was carried out by means of a paired-sample *t*-test. The amplitude of the N2pc wave was significantly larger on the hemisphere contralateral to the hemifield in which a Gestalt configuration was presented, compared with the hemisphere contralateral to the hemifield in which a non-Gestalt was simultaneously presented [*t*(14) = 2.03, *p* = 0.03, *d*_z_ = 0.62] (**Figure 3**). Because the N2pc is a *contralateral minus ipsilateral* difference wave with a PO distribution (see scalp map in **Figure 3**), this finding indicates that Gestalt configurations elicited enhanced N2 amplitudes when presented in either hemifield simultaneously with a non-Gestalt stimulus in the opposite hemifield.

#### Across-Trial Analysis of Gestalt Configurations (GG and nn Trials)

##### P1

**FIGURE 4:**
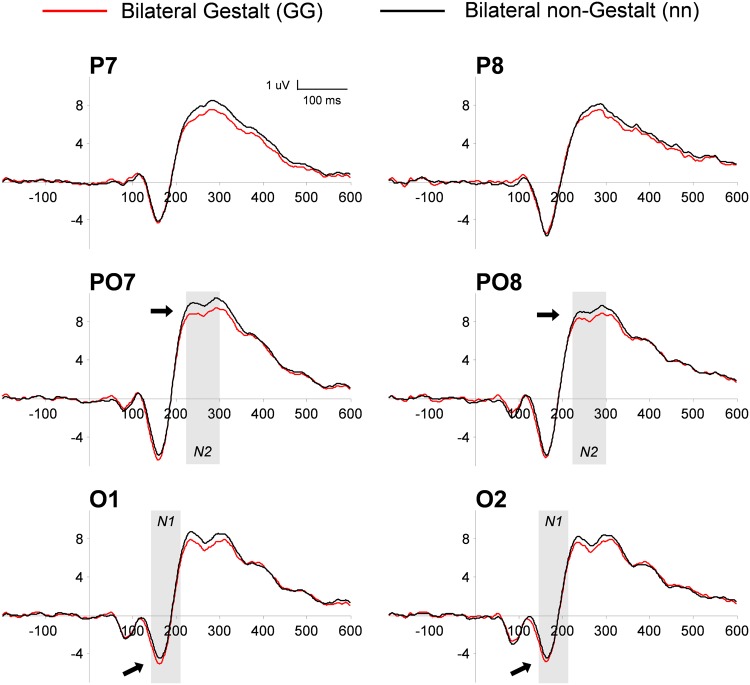
**Grand-average of PO ERP waveforms for bilateral Gestalt and non-Gestalt stimuli.** Average ERP waveforms throughout the epochs (-200 to 600 ms) for bilateral Gestalt (red line) and bilateral non-Gestalt (black line) stimuli at occipital (O), PO, and parietal (P) electrodes on both hemispheres. Arrows indicate significant effects on N1 and N2 components and the shaded gray areas indicate time-windows in which significant effects were found.

This analysis used a three-way ANOVA factoring Electrode Location (occipital, PO, parietal), Hemisphere (left, right), and Gestalt Presence (present, absent). The electrode location interacted with the presence (vs. absence) of a Gestalt configuration [*F*(2,28) = 5.59, *p* < 0.01, η^2^= 0.28], corresponding to a positive-signed difference for Gestalt minus non-Gestalt stimuli on parietal electrodes that reverted its sign becoming negative-signed on occipital electrodes [Gestalt minus non-Gestalt on P versus O electrodes: *t*(15) = 2.81, *p* = 0.01, *d_z_* = 0.73]. Further exploration of this interaction by means of paired *t*-tests on each electrode pair (left versus right) did not reveal any significant difference in the P1 range between stimuli with and without Gestalt configurations [O electrodes: *t*(15) = 1.29, *p* = 0.21; PO electrodes: *t*(15) = 1.06, *p* = 0.31; P electrodes: *t*(15) = 0.7, *p* = 0.94] (**Figure 4**).

##### N1

This analysis used a three-way ANOVA factoring Electrode Location (occipital, PO, parietal), Hemisphere (left, right), and Gestalt Presence (present, absent). Similarly to what we observed in the P1 range, the electrode location interacted with the presence (vs. absence) of a Gestalt configuration [*F*(2,28) = 10.8, *p* < 0.001, η^2^= 0.44]. Further analysis of this interaction by means of paired *t*-tests revealed a trend for a larger amplitude of the occipital N1 for bilateral Gestalt configurations compared to bilateral non-Gestalt configurations [*t*(14) = 2.15, *p* = 0.05, *d_z_* = 0.55] (**Figure 4**). No such effect was found at PO [*t*(14) = 0.8, *p* = 0.44] and at parietal electrodes [*t*(14) = 1.02, *p* = 0.33].

##### N2

This analysis used a three-way ANOVA factoring Electrode Location (occipital, PO, parietal), Hemisphere (left, right), and Gestalt Presence (present, absent). A main effect of Electrode Location was found, indicating that the largest amplitude of the N2 component was observed over PO electrodes [*F*(2,28) = 7.45, *p* < 0.01, η^2^= 0.35]. Moreover, the N2 was more negative for Gestalt (vs. non-Gestalt) configurations across all electrode locations and hemispheres (**Figure 4**), as indicated by a significant main effect of Gestalt Presence [*F*(1,14) = 5.36, *p* < 0.05, η^2^= 0.28].

##### P3

**FIGURE 5:**
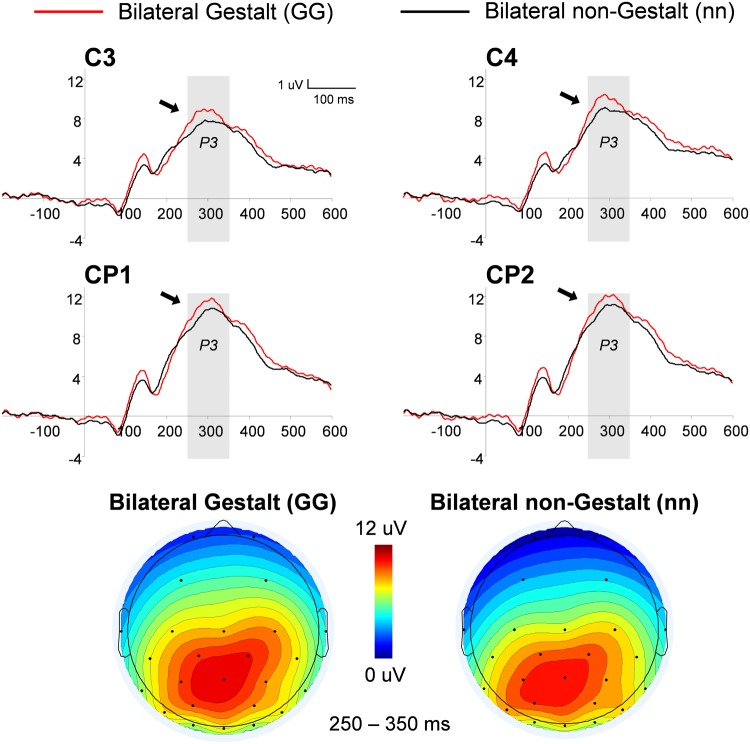
**Grand-average and voltage maps of centro-parietal (CP) ERP waveforms for bilateral Gestalt and non-Gestalt stimuli.** Average ERP waveforms throughout the epochs (-200–600 ms) for bilateral Gestalt (red line) and bilateral non-Gestalt (black line) stimuli at central (C) and CP electrodes on both hemispheres. Arrows indicate significant effects for the P3 component and the shaded gray areas indicate time-windows in which significant effects were found. Maps on the bottom show the average voltage maps relative to the bilateral Gestalt (left) and bilateral non-Gestalt (right) conditions within the 250–350 ms time-window.

This analysis used a three-way ANOVA factoring Electrode Location (centro-parietal (CP), central), Hemisphere (left, right), and Gestalt Presence (present, absent). An overall increase in the P3 amplitude was observed for Gestalt (Versus non-Gestalt) bilateral configurations [*F*(1,14) = 6.85, *p* < 0.05, η^2^= 0.33] (**Figure 5**). Additionally, mean amplitudes were overall larger in the right (vs. left) hemisphere, for both Gestalt and non-Gestalt stimuli [*F*(1,14) = 40.4, *p* < 0.01, η^2^= 0.74].

## Discussion

In this study, we provided electrophysiological evidence supporting the notion that the human brain differentiates responses to visual stimuli with Gestalt configurations from physically matched stimuli without Gestalt configurations starting at about 150 ms post-stimulus.

The N2pc component reflects the attentional selection of a lateralized target stimulus when such stimulus competes for attentional resources with a non-target stimulus simultaneously displayed in the opposite visual hemifield (e.g., Luck and Hillyard, 1994). Only a few studies have used the N2pc to study automatic processes of attention to Gestalt stimuli (Conci et al., 2006, 2011; Töllner et al., 2015; Wiegand et al., 2015), while other studies were not appropriate for studying the N2pc either because non-lateralized visual stimuli were used (Han et al., 2005) or because sensory information was not balanced across hemifields (Kasai et al., 2011). Both requirements need to be satisfied in the design of a proper N2pc study. Previous research found limited ERP effects related to Gestalt organization for stimuli outside the scope of selective attention (Han et al., 2005). In contrast, we believe that the N2pc design used here may be more sensitive to investigate automatic attentional processes. Typically, N2pc paradigms explicitly define what stimuli represent targets with respect to non-targets. In such experimental circumstances, the N2pc may signal the match of the perceived visual stimulus with an existing top-down target template or with a currently active task-set (Eimer and Kiss, 2008; Kiss et al., 2008; Mazza et al., 2009; reviewed in Luck, 2011). We designed our study with the intention to avoid the involvement of any top-down attentional templates or stimulus-specific task-sets. In the current paradigm, notably, there was no distinction between targets and non-targets as participants were required to respond as quickly as possible when *any* visual stimulus appeared on display – regardless of whether it contained one, two, or no Gestalt configurations (importantly, the possible presence of Gestalt configurations was never mentioned in task instructions). This represents a crucial difference relative to previous N2pc studies of Gestalt perception with task designs that required target selection (e.g., Conci et al., 2006). Therefore, any N2pc effect reflecting a preferential orienting toward a specific type of stimulus is likely to be truly automatic and related to stimulus-driven attentional capture (Hickey et al., 2006, 2009). Our results on the N2pc suggest that between 220 and 300 ms after stimulus onset a bottom-up attentional signal takes place contralateral to the visual stimuli with a Gestalt configuration, presumably as a result of their intrinsic saliency (Girelli and Luck, 1997). Thus, Gestalt configurations may be inherently salient, similarly – for example – to visual motion, which is the only other visual feature that has been shown to elicit a robust N2pc even when task-irrelevant or when top-down attention was directed elsewhere (Girelli and Luck, 1997). Interestingly, the N2pc has been observed not only when attention is deployed toward specific spatial locations, but also when it is directed toward objects irrespective of their spatial location (Woodman et al., 2009). Moreover, a recent study identified an enhanced N2pc – indicating automatic attentional capture – contralateral to objects that were implicitly associated with rewards, thus confirming that the N2pc is sensitive to automatic attentional modulations by objects with salient properties (Donohue et al., 2016). In our design, Gestalt configurations were characterized by both a specific spatial location and an object-like configuration, therefore the N2pc enhancement for Gestalt configurations observed in this study may reflect space-based attentional capture, object-based capture, or a combination of both.

An issue that needs to be discussed concerns the nature of the effects observed in this study. Previous work has shown that Gestalt grouping principles may operate at a very early processing stage and independently from the engagement of attention (Conci et al., 2007, 2011; Poscoliero et al., 2013). This work differed from our study because it used illusory figures as stimuli; nonetheless one may still wonder whether the effects we found here are related to pre-attentive or to attentive processes. Pre-attentive processing is directed holistically toward the entire visual scene (Treisman, 1986) and typically occurs within the first 100 ms of stimulus processing (Theeuwes, 1995; Woodman and Luck, 2003). Importantly, we did not observe any lateralized ERP modulation before the N2pc, which occurs at a later processing stage and has been associated with spatial and object-based attention processes (Woodman and Luck, 2003; Woodman et al., 2009). Therefore, the ERP modulations described here likely track allocation of attention. This does not necessarily imply that pre-attentive processes were not at play in our task. Rather, even though the present study was focused on the automatic attentional capture by Gestalt stimuli, such capture could possibly arise after a pre-attentive processing stage. Although we did not identify early ERP signatures of pre-attentive processing of Gestalt stimuli, we acknowledge that other task-designs, such as those with illusory figures and search or choice paradigms, are probably more suitable to identify pre-attentive ERP correlates of Gestalt processing (e.g., Conci et al., 2007, 2011; Poscoliero et al., 2013).

In order to characterize the psychophysiological processing of Gestalt configurations at various temporal processing stages, we conducted analyses on several other ERP components. No significant effect emerged on the P1, i.e., a component which reflects perceptual processing of visual stimuli occurring in the striate and extrastriate cortex and whose amplitude may be modulated by top-down attention (Woldorff et al., 2002; Di Russo et al., 2003). In agreement with our predictions, this negative result – although it should be taken with caution (e.g., Poldrack, 2006) – indicates that bilateral Gestalt configurations do not entail special perceptual processing in a simple detection task with no top-down manipulation of visual attention. Notably, Gestalt and non-Gestalt visual stimuli were matched for their physical attributes (yet they were not always matched for internal symmetry) and therefore the Gestalt-related effects found on the N1, N2, and N2pc components do not reflect differences in the basic physical attributes of the stimuli.

Starting at about 150 ms post-stimulus, we observed a larger mean amplitude of the N1 in the presence of bilateral Gestalt configurations relative to bilateral non-Gestalt configurations. Previous research has demonstrated that unconnected perceptual units that are grouped together according to Gestalt principles yield an increased visual N1 (Kasai and Kondo, 2007; Kasai et al., 2011). These findings are in line with existing evidence demonstrating enhanced brain responses in the lateral occipital complex (LOC) for stimuli that give rise both to contours and to real or illusory shapes (Stanley and Rubin, 2003; Altmann et al., 2003; Kourtzi et al., 2003). This interpretation is in line with recent results showing that the spatial grouping of discrete visual elements into a global pattern enhance ERP amplitudes in the ventrolateral extrastriate cortex (Montoro et al., 2015). In keeping with that, we observed an increased N1 amplitude in response to visual stimuli whose spatial configuration leads to their perceptual grouping into closed shapes. Moreover, we observed a greater negativity for bilateral non-Gestalt relative to bilateral Gestalt configurations in the N2 component around 250 ms after stimulus onset. The N2 observed in this study has a relatively small absolute amplitude and appears to be riding a positive waveform, possibly the P2 (Luck et al., 2000). Alternatively, as a reviewer suggested, the N2 may simply represent the opposite side of the P3 dipole; however, inspection of the time-course and topography of the N2 and of the P3 seems to rule out this possibility. Nonetheless, the greater negativity in the N2 time-range likely represents a continuation of the N1 effect and is in agreement with previous ERP results with Gestalt stimuli (Kasai et al., 2011).

Furthermore, we observed an increased amplitude in the CP P3 for bilateral Gestalt stimuli relative to bilateral non-Gestalt stimuli. The P3 component has been suggested to include two sub-components, a fronto-centrally distributed P3a and a centro-parietally distributed P3b. The latter, whose topographical localization resembles that found in this study, has been related to mechanisms of attentional orienting (reviewed in Polich, 2007). Complementing the evidence for automatic attentional capture by Gestalt stimuli suggested by our N2pc findings, the P3 increase observed on bilateral Gestalt trials may reflect mechanisms of attentional orienting toward stimuli with increased saliency due to their Gestalt configurations.

In this study we did not observe any behavioral effect of Gestalt stimuli, whereas a potential speeding-up of RTs might have been expected. However, it is worth underlying that we used a simple reaction time task in which subjects were instructed to respond as soon as they saw any stimulus. Therefore, the perceptual and attentional analysis of the visual stimuli was not necessary to initiate the response processes. For this reason, it appears plausible that no behavioral differences are observed given the nature of the task, which is inherently different from other types of tasks involving stimulus discrimination, target-template matching, and/or forced-choice responses. Actually, the very fact that despite the lack of a behavioral difference there was a differential effect on ERPs shows that stimulus displays elicit an automatic capture of attention when there are good Gestalt parts. This capture is witnessed neurophysiologically but does not necessarily end up with a difference in the motor output given that the task does not require a differential response for Gestalt and no-Gestalt stimuli. Yet, it might represent the neural correlate of a preparation for a potential differential motor response.

Taken together, our results indicate that visual objects organized according to Gestalt principles are prioritized over competing non-Gestalt stimuli in a bottom-up and automatic fashion, as indicated by the N2pc effect, and are associated with augmented cognitive processing, as indicated by the greater amplitude of the N1 and P3 components. A commonly accepted framework is that Gestalt grouping occurs pre-attentively (Rauschenberger and Yantis, 2001; Russell and Driver, 2005; Lamy et al., 2006; Conci et al., 2007, 2011; Müller et al., 2010), although task demands might overcome grouping, for example when object history indicates an advantage of maintaining separate representations (Luria and Vogel, 2014). Complementarily, here we showed that even without any strong task demands or top-down template, Gestalt grouping is associated with automatic stimulus-driven attentional capture.

## Conclusion

The present study characterized the electrophysiological responses evoked by Gestalt-based visual configurations and demonstrated that the stimulus organization based on Gestalt principles entails an automatic attentional capture and characteristic psychophysiological signatures starting at about 150 ms after stimulus onset.

In addition to their general contribution to understanding the neural correlates of visual perception, these results encourage the use of Gestalt-organized stimuli to improve the rehabilitative treatment of patient with cortically or sub-cortically impaired visual or attentional functions such as visuo-spatial neglect or hemianopia (Celeghin et al., 2015; Georgy et al., 2016).

## Author Contributions

Both authors devised the rationale and the experimental paradigm of the study. FM programmed the paradigm and performed data collection and analysis under the supervision of CM. FM drafted the manuscript and CM provided critical revisions. Both authors approved the final version of the manuscript for submission.

## Conflict of Interest Statement

The authors declare that the research was conducted in the absence of any commercial or financial relationships that could be construed as a potential conflict of interest.
